# Short-term outcomes among patients with subclinical hypothyroidism undergoing primary percutaneous coronary intervention

**DOI:** 10.4314/gmj.v57i1.6

**Published:** 2023-01

**Authors:** Abdulameer J al-Gburi, Saba R al-Obaidi, Wasnaa H Abdullah

**Affiliations:** 1 Department of Medicine, College of Medicine, al-Mustansiriyah University, Baghdad, Iraq; 2 Department of Obstetrics and Gynecology, College of Medicine, al-Nahrain University, Baghdad, Iraq; 3 Department of Pediatrics, College of Medicine, al-Mustansiriyah University, Baghdad, Iraq

**Keywords:** Primary percutaneous coronary intervention, thyroid function test, ST-elevation myocardial infarction, subclinical hypothyroidism

## Abstract

**Objectives:**

This study aimed to examine possible associations between previously undiagnosed subclinical hypothyroidism and short-term outcomes and mortality in a sample of Iraqi patients undergoing primary percutaneous coronary intervention for ST-segment elevation myocardial infarction.

**Design:**

This is a prospective observational cohort study.

**Setting:**

The study was conducted in a single tertiary referral centre in Baghdad, Iraq.

**Participants:**

Thyroid-stimulating hormone and free T4 levels were measured in 257 patients hospitalised with ST-elevation myocardial infarction who underwent primary percutaneous coronary intervention between January 2020 and March 2022.

**Main outcome measures:**

Adverse cardiovascular and renal events during hospitalisation and 30-day mortality were observed.

**Results:**

Previously undiagnosed subclinical hypothyroidism was detected in 36/257 (14%) ST-elevation myocardial infarction patients and observed more commonly in females than males. Patients with subclinical hypothyroidism had significantly worse short-term outcomes, including higher rates of suboptimal TIMI Flow (< III) (p =0.014), left ventricular ejection fraction ≤ 40% (p=0.035), Killip class >I (p=0.042), cardiogenic shock (p =0.016), cardiac arrest in the hospital (p= 0.01), and acute kidney injury (p= 0.044). Additionally, 30-day mortality was significantly higher in patients with subclinical hypothyroidism (p= 0.029).

**Conclusion:**

Subclinical hypothyroidism previously undiagnosed and untreated had a significant association with adverse short-term outcomes and higher short-term mortality within 30 days compared to euthyroid patients undergoing primary percutaneous coronary intervention. Routine thyroid function testing during these patients' hospitalisation may be warranted.

**Funding:**

None declared

## Introduction

Subclinical hypothyroidism (SCH) is defined biochemically as a normal serum free thyroxine (T4) level in the presence of an increased serum thyroid-stimulating hormone (TSH) concentration.^1^ Its prevalence ranges from 4 to 15 per cent and is higher in females and increasing age.^2^ Overt hypothyroidism was associated with accelerated atherosclerosis and an increased risk of cardiovascular abnormalities. 3 These patients also experienced more adverse outcomes after percutaneous coronary intervention (PCI).^4^

Some studies have reported a higher atherosclerotic cardiovascular disease risk in patients with SCH. 5–8 Elevated TSH levels were observed to be associated with higher cholesterol levels.^9^ Higher mortality was also reported in some studies^6^,^10^, especially with TSH ≥ 10.0 mIU/L, in contrast to other studies.^11^,^12^ Heart failure events and myocardial infarction have been reported to be higher.^13^,^14^

These findings in SCH patients could be explained by mitochondrial oxidative stress due to elevated inflammatory markers, hypercoagulability, endothelial dysfunction, insulin resistance, increased vascular resistance and left ventricular diastolic and systolic dysfunction.^3^,^15^,^16^

As is the case with overt hypothyroidism, SCH was observed to be associated with elevated peripheral vascular resistance and diastolic dysfunction.^17^ There are a few studies evaluating the effects of subclinical hypothyroidism on the outcomes of ST-segment elevation myocardial infarction (STEMI) patients in the modern era of mechanical reperfusion, especially in developing countries like Iraq. Also, this study includes some data not included in previous studies.

This study aimed to examine possible associations between previously undiagnosed subclinical hypothyroidism and short-term outcomes and mortality in a sample of Iraqi patients undergoing primary percutaneous coronary intervention (PPCI) for STEMI.

## Methods

**Figure 1:**
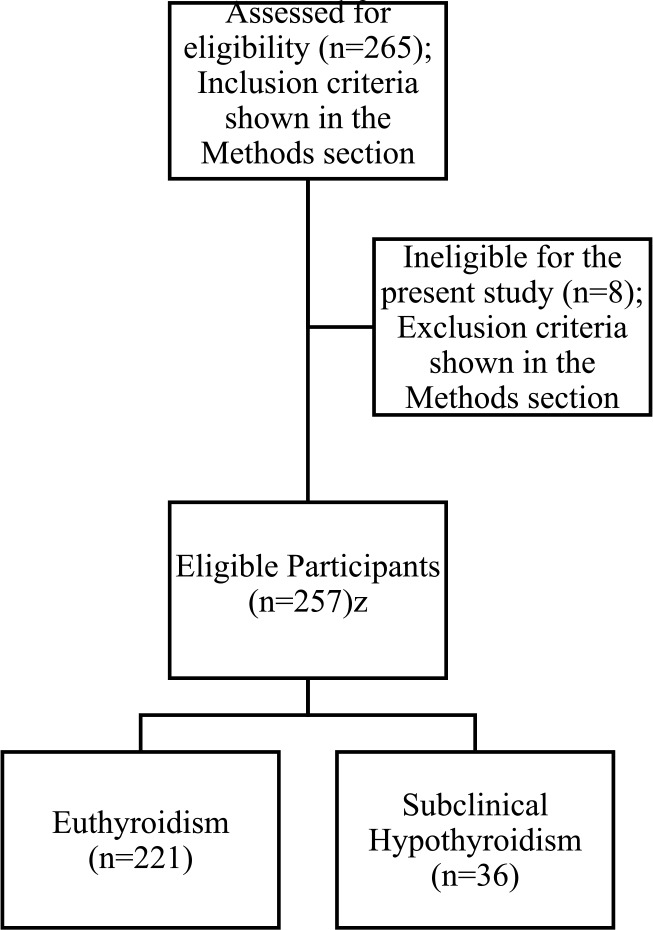
Flow chart of the study sample selection

This prospective observational study was conducted at a tertiary referral centre in Baghdad, Iraq (researcher institution), which provided primary PCI services 24 hours a day/seven days a week. This study includes STEMI patients admitted between January 2020 and March 2022 who underwent primary PCI. Excluded were patients taking amiodarone, having a history of hyperthyroidism and hypothyroidism, or taking treatment for thyroid dysfunction. Patients with rheumatoid arthritis, untreated adrenal insufficiency, and Class II or III obesity were excluded as these may affect TSH levels. The Cochran formula [*n* = Zα 2pq/d^2^] was used to calculate the minimum sample size, n 18; where Zα is the standard deviation of the normal distribution, given as 1.96 at a significance level of 0.05; p is the percentage to be assessed in the population, taken as the percentage of subclinical hypothyroidism (15 per cent)^19^–^21^; q=1-p; d represents the acceptable error margin, which is set at 5 per cent. n has been calculated to equal 196. Assuming a non-responder rate of 30 per cent, the final sample size was estimated to be 255. The flow chart of the study sample selection is shown in Figure 1.

STEMI was diagnosed following established protocols, including chest pain, electrocardiographic findings, and biomarker changes.^22^ PPCI was performed when symptoms started less than 12 hours and up to 24 hours with continuous chest pain at admission.^22^ The duration of symptoms was identified as the period between the symptom onset and admission to the cardiac centre. Door to Balloon time was identified as the period between admission to the cardiac centre to the first balloon inflation during PCI.

Demographic characteristics at baseline, cardiovascular history, risk factors for cardiovascular disease, and laboratory tests (TSH, free T4, haemoglobin, Glycated Hemoglobin (A1C), serum creatinine, and lipid indices) data were collected from enrolled patients. The normal ranges of thyroid function tests were 0.40–4.99 mIU/L for TSH and 0.7–1.8 ng/dL for free T4. The same testing method was performed on all samples from all patients. A TSH concentration equal to or more than 5 mU/mL with a normal free T4 level was defined as SCH.^1^ The Chronic Kidney Disease-Epidemiology Collaboration (CKD-EPI) equation was used to calculate the Glomerular Filtration Rate (GFR).^23^ According to the Kidney Disease: Improving Global Outcomes (KDIGO) criteria, acute kidney injury (AKI) was defined as either a rise in serum creatinine of more than 0.3 mg/dL in less than 48 hours of admission or a rise in serum creatinine more than 1.5 times the baseline level within the previous seven days.^24^

Short-term complications and events during hospitalisation were documented, and patients were followed up for 30 days. The documented in-hospital events include cardiogenic shock, Ejection fraction less than 40%, Killip class more than one^25^, new atrial fibrillation, sudden cardiac arrest, bradyarrhythmia necessitating pacing, major bleeding necessitating blood transfusion, and acute kidney injury while hospitalised. Echocardiography was performed within 24 hours. Thrombolysis in Myocardial Infarction (TIMI) risk score for ST-elevation myocardial infarction was caculated^26^. Coronary angiography and PCI data were collected, including any placement of stents, the number of diseased vessels, culprit vessel, post-PCI TIMI flow, and contrast volume used. No intra-coronary imaging (IVUS or OCT) was used. Revascularisation was done only for the culprit lesions. The medications list at discharge was documented. All processes were carried out per ethical standards, approved by the Local Ethics Committee in the researcher institution (REG0101/2020) and adhered to the 1964 Declaration of Helsinki and its subsequent amendments. Written informed consent was obtained from all participants in this study.

### Data Analysis

Continuous variables are normally distributed, expressed as means with standard deviations, and compared using an independent sample t-test. The medians and interquartile ranges were calculated for continuous variables that are non-normally distributed, and the Mann-Whitney U test was used to compare them. Numbers and percentages represent categorical variables, and the chi-square test was used to calculate the p-Values. For all analyses, significance was defined as a two-tailed p-Value of 0.05. Statistical Package for the Social Sciences (SPSS) version 26.0 for Windows was used for all analyses (SPSS Inc., Chicago, IL, USA).

## Results

### Baseline Characteristics

A total of 257 patients treated with primary PCI after being diagnosed with STEMI were recruited during the study duration. The mean age was 57 years, 27% were females, and 36 (14%) were observed to have SCH. Table 1 compares the two groups' clinical and laboratory characteristics at baseline and the medications prescribed at discharge. Females were more frequently diagnosed with SCH (p-Value=0.015).

**Table 1:** Baseline characteristics of the study population

	All Patients (n=257)	Euthyroidism (n = 221)	Subclinical Hypothyroidism (n = 36)	p-Value
History and Examinations findings				
**Age, years, mean ± SD**	57.82±8.92	57.70±8.65	58.54±10.51	0.605
**Older patients (> 70 years), n (%)**	26(10.1)	20(9.0)	6(16.7)	0.160
**Female sex, n (%)**	71(27.6)	55(24.9)	16(44.4)	**0.015**
**Hypertension, n (%)**	131(51.0)	112(50.7)	19(52.8)	0.815
**Diabetes mellitus, n (%)**	70(27.2)	56(25.3)	14(38.9)	0.090
**Smoker, n (%)**	95(37.0)	81(36.7)	14(38.9)	0.796
**Previous MI, n (%)**	20(7.8)	17(7.7)	3(8.3)	0.894
**Previous PCI, n (%)**	25(9.7)	20(9.0)	5(13.9)	0.364
**Previous stroke, n (%)**	17(6.6)	15(6.8)	2(5.6)	0.783
**Symptoms onset to admission,** **minutes, median [IQR 25–75]**	334.09[208.73–455.35]	334.50 [218.13–455.35]	303.73 [183.75–456.70]	0.695
**Door to Balloon, minutes, median** **[IQR 25–75]**	137.48[103.58–175.22]	138.60[103.59–173.95]	129.42[98.19–185.03]	0.805
**BMI, kg/m^2^, mean ± SD**	25.31±5.53	25.11±5.28	26.59±6.83	0.137
Laboratory findings, **mean ± SD**				
**Hb, g/dL**	13.57±2.03	13.62±1.99	13.24± 2.23	0.292
**eGFR (ml/min/1.73 m^2^)**	82.76±24.72	82.10±25.21	86.82±21.29	0.289
**A1C, %**	5.85±1.85	5.83±1.93	6.00±1.31	0.596
**TC, mg/dL**	173.07±40.15	172.18±41.26	178.55±32.50	0.379
**LDL, mg/dL**	105.26±26.60	104.79±26.65	108.13±26.46	0.485
**HDL, mg/dL**	39.47±10.93	39.42±10.76	39.80±12.08	0.846
**TG, mg/dL**	137.67±33.73	136.90±33.11	142.42±37.47	0.363
Medications after discharge, **n (%)**				
**Aspirin**	254(98.8)	219(99.1)	35(97.2)	0.332
**Clopidogrel**	85(33.1)	69(31.2)	16(44.4)	0.118
**Ticagrelor**	168(65.4)	149(67.4)	19(52.8)	0.087
**β-Blocker**	241(93.8)	208(94.1)	33(91.7)	0.572
**ACEI/ARB**	218(84.8)	190(86.0)	28(77.8)	0.204
**Statins**	254(98.8)	219(99.1)	35(97.2)	0.332

**Abbreviations:** n stands for Number; SD stands for Standard Deviation; MI stands for myocardial infarction; PCI stands for Percutaneous Coronary Intervention; IQR stands for Interquartile Range; BMI stands for body mass index; Hb stands for Haemoglobin level; eGFR stands for estimated Glomerular Filtration Rate; A1C stands for Glycated Haemoglobin; TC stands for Total Cholesterol; HDL stands for High-Density Cholesterol; LDL stands for Low-Density Cholesterol; TG stands for Triglycerides; ACEI/ARB stands for angiotensin-converting enzyme inhibitors/angiotensin-receptor blockers; Significance is denoted by bold font.

### Coronary Angiography and PCI Outcomes

No significant difference was observed between the two groups regarding the number of diseased vessels and the culprit vessel during coronary angiography.

However, suboptimal TIMI Flow (< III) was more prevalent in patients with SCH (p-Value=0.014), with no significant difference in the percentage of patients undergoing stenting and contrast volume used, as shown in Table 2.

**Table 2:** Procedural features of the study population

	Euthyroidism (n = 221)	Subclinical Hypothyroidism (n = 36)	p-Value
**Number of diseased arteries, n (%)**			
1	56(25.3)	11(30.6)	0.509
2	79(35.7)	12(33.3)	0.779
3	86(38.9)	13(36.1)	0.749
**Culprit vessel, n (%)**			
**LAD**	115(52.0)	19(52.8)	0.934
**LCX**	46(20.8)	9(25.0)	0.570
**RCA**	60(27.1)	8(22.2)	0.534
Post-PCI TIMI < III, n (%)	12(5.4)	6(16.7)	**0.014**
Stenting, n (%)	197(89.1)	31(86.1)	0.594
Contrast Volume, mL	246.34±126.81	260.99±142.72	0.529

**Abbreviations:** n, Number; LAD stands for Left Anterior Descending; RCA stands for Right Coronary Artery; LCx stands for Left Circumflex; TIMI stands for Thrombolysis In Myocardial Infarction; Significance is denoted by bold font.

### Short-term Outcomes and Mortality

The short-term outcomes of patients with SCH in comparison to euthyroid patients are summarised in Table 3. Participants diagnosed with SCH were likelier to have higher TIMI risk scores, reduced Ejection Fraction ≤ 40%, Killip class >I, cardiogenic shock, cardiac arrest in hospital, and acute kidney injury. Both groups demonstrate no significant difference in the development of bradyarrhythmia requiring a pacemaker, new atrial fibrillation, major bleeding, and the length of stay in the hospital. Four out of 36 (11.1%) patients with SCH died within 30 days of hospitalisation, compared to 7/221 (3.2%) euthyroid patients (p=0.029).

**Table 3:** Short-term outcomes and mortality

Variable	Euthyroidism (n = 221)	Subclinical Hypothyroidism (n = 36)	p- Value
**TIMI risk score, median [IQR** **25–75]**	4.80[4.19–6.08]	5.86[4.51–7.10]	**0.043**
**Ejection Fraction ≤ 40%, n (%)**	60(27.1)	16(44.4)	**0.035**
**Killip class >I, n (%)**	41(18.6)	12(33.3)	**0.042**
**Cardiogenic shock, n (%)**	6(2.7)	4(11.1)	**0.016**
**Cardiac arrest in hospital, n** **(%)**	3(1.4)	3(8.3)	**0.010**
**Bradyarrhythmia, n (%)**	4(1.8)	2(5.6)	0.168
**Atrial fibrillation (New), n (%)**	3(1.4)	1(2.8)	0.523
**Bleeding, n (%)**	10(4.5)	2(5.6)	0.786
**Acute Kidney Injury, n (%)**	23(10.4)	8(22.2)	**0.044**
**Hospitalisation, days, mean ±** **SD**	4.10(5.03)	4.76(6.51)	0.489
**Mortality (30 Days), n (%)**	7(3.2)	4(11.1)	**0.029**

**Abbreviations**: n stands for Number; TIMI stands for Thrombolysis in Myocardial Infarction, and statistical significance is denoted by bold font.

## Discussion

This prospective observational research was done in a single cardiac centre. Previously undiagnosed and untreated SCH was found to have a significant association with adverse short-term outcomes and higher short-term mortality within 30 days compared to euthyroid subjects in patients undergoing PPCI.

A meta-analysis of seven studies found increased coronary heart disease risk with increasing serum TSH levels, especially with TSH levels of more than 10 mU/L.^27^ In a large cross-sectional study, elevated TSH level was associated with significantly higher total cholesterol levels than euthyroid individuals.^2^

An analysis of six cohort studies found a significant increase in heart failure events with increasing TSH levels.^13^ Additionally, a meta-analysis involving patients with heart failure with decreased ejection fraction found that those with SCH had a higher risk of all-cause mortality than those with euthyroidism.^28^ There are contradictory findings regarding the effect of SCH on mortality. Cardiovascular mortality, but not all-cause mortality, was found to be increased with increasing TSH levels in a meta-analysis of eleven cohort studies. 27

Individuals with SCH have a higher risk of adverse outcomes following PCI than those who are euthyroid, which is particularly relevant to our study.^29^,^30^ Following ST-elevation myocardial infarction, a study concluded that these patients had significantly worse outcomes, including increased left ventricular systolic dysfunction, acute kidney injury, and 30-day mortality.^31^ Moreover, long-term mortality was also significantly higher.^31^–^33^ In contrast to these findings, another study found no difference in LV ejection function among individuals with the acute coronary syndrome.^34^

In the current study, SCH was associated with a higher risk of acute kidney injury than euthyroid patients. These results agree with many studies, including cardiovascular disease patients. 35 In a recent study, when age-specific rather than uniform normal reference ranges were used in individuals over 70, the prevalence of SCH decreased from 29.6 to 3%.^36^ Our study found no significant difference in the proportion of those older than 70, which precludes possible bias.

The thyroid replacement therapy benefit for patients with SCH is debatable, and none were put on thyroid replacement therapy. In younger SCH subjects (aged 40–70 years) treated with thyroid replacement therapy, a lower incidence of coronary heart disease was observed, and this finding was not observed in older (aged over 70 years) patients.^37^

This study is a non-randomised observational study done in a single centre. The sample size of SCH patients is small. Residual confounding by unmeasured variables cannot be ruled out. Additionally, because this study included only patients undergoing PPCI, the findings cannot be generalised to all patients with STEMI. Information on past medications and further diagnostics like antithyroid peroxidase (anti-TPO) antibodies level and autoantibodies to TSH (causing “macro-TSH) were not measured in this study.

## Conclusion

Undiagnosed SCH is common among STEMI patients undergoing PPCI and may be used as a predictor for adverse short-term outcomes and mortality. These research results have significant clinical implications because STEMI and SCH are more prevalent as the population ages.
